# Supplementary material for: End Sequence Analysis Toolkit (ESAT) expands the extractable information from single-cell RNA-seq data
Source: Genome Res. 2016 Oct;26(10):1397–410. doi: 10.1101/gr.207902.116 (PMC5052061; doi:10.1101/gr.207902.116)
Supplement: Supplemental Material [file supp_gr.207902.116_Supp_Materials.tar.gz › ESAT-esat_v0.1/doc/html/classbroad_1_1pda_1_1seq_1_1segmentation_1_1_continuous_data_alignment_model-members.html]

ESAT: Member List


ESAT


- Main Page
- Related Pages
- Packages
- Classes
- Files

- Class List
- Class Index
- Class Hierarchy
- Class Members

All Classes Namespaces Files Functions Variables Enumerator Pages

- broad
- pda
- seq
- segmentation
- ContinuousDataAlignmentModel

broad.pda.seq.segmentation.ContinuousDataAlignmentModel Member List

This is the complete list of members for broad.pda.seq.segmentation.ContinuousDataAlignmentModel, including all inherited members.

|  |  |  |
| --- | --- | --- |
| calculatePVal(int k, double lambda, double w, double T) | broad.pda.seq.segmentation.ContinuousDataAlignmentModel | static |
| ContinuousDataAlignmentModel(AlignmentDataModel data) | broad.pda.seq.segmentation.ContinuousDataAlignmentModel |  |
| ContinuousDataAlignmentModel(AlignmentDataModelStats data) | broad.pda.seq.segmentation.ContinuousDataAlignmentModel |  |
| ContinuousDataAlignmentModel(AlignmentDataModelStats data, int EF, int minSpliceSupport) | broad.pda.seq.segmentation.ContinuousDataAlignmentModel |  |
| ContinuousDataAlignmentModel(AlignmentDataModelStats data, File[] maskFiles, int minSpliceSupport) | broad.pda.seq.segmentation.ContinuousDataAlignmentModel |  |
| ContinuousDataAlignmentModel(AlignmentDataModelStats data, File[] maskFiles, int EF, int minSpliceSupport, AlignmentDataModelStats pairedData, AlignmentDataModelStats strandSpecificReads) | broad.pda.seq.segmentation.ContinuousDataAlignmentModel |  |
| ContinuousDataAlignmentModel(AlignmentDataModelStats data, File[] maskFiles, int EF, int minSpliceSupport, AlignmentDataModelStats pairedData, AlignmentDataModelStats strandSpecificReads, boolean upweight) | broad.pda.seq.segmentation.ContinuousDataAlignmentModel |  |
| ContinuousDataAlignmentModel(AlignmentDataModelStats data, Map< String, Integer > maskFileData, int EF, int minSpliceSupport, AlignmentDataModelStats pairedData, AlignmentDataModelStats strandSpecificReads) | broad.pda.seq.segmentation.ContinuousDataAlignmentModel |  |
| ContinuousDataAlignmentModel(AlignmentDataModelStats data, Map< String, Integer > maskFileData, int EF, int minSpliceSupport, AlignmentDataModelStats pairedData, AlignmentDataModelStats strandSpecificReads, boolean upWeight) | broad.pda.seq.segmentation.ContinuousDataAlignmentModel |  |
| ContinuousDataAlignmentModel(AlignmentDataModelStats data, Map< String, Integer > maskFileData, int EF, int minSpliceSupport, AlignmentDataModelStats pairedData, AlignmentDataModelStats strandSpecificReads, int chunkSize, boolean upWeightSplices) | broad.pda.seq.segmentation.ContinuousDataAlignmentModel |  |
| ContinuousDataAlignmentModel(AlignmentDataModelStats data, File[] maskFiles, int EF, int minSpliceSupport, AlignmentDataModelStats pairedData) | broad.pda.seq.segmentation.ContinuousDataAlignmentModel |  |
| ContinuousDataAlignmentModel(AlignmentDataModelStats data, File[] maskFiles, int EF, int minSpliceSupport) | broad.pda.seq.segmentation.ContinuousDataAlignmentModel |  |
| count(Annotation align) | broad.pda.seq.segmentation.ContinuousDataAlignmentModel |  |
| CV(Gene gene, IntervalTree< Alignment > tree) | broad.pda.seq.segmentation.ContinuousDataAlignmentModel |  |
| DEFAULT\_INS\_SIZE\_PVAL | broad.pda.seq.segmentation.ContinuousDataAlignmentModel | static |
| DEFAULT\_INSERT\_SIZE\_FUDGE | broad.pda.seq.segmentation.ContinuousDataAlignmentModel | static |
| DEFAULT\_MAKE\_GENE\_OVERLAP | broad.pda.seq.segmentation.ContinuousDataAlignmentModel | static |
| DEFAULT\_MIN\_MAPPING\_QUALITY | broad.pda.seq.segmentation.ContinuousDataAlignmentModel | static |
| filterGenesByExpression(Map< Gene, List< Double >> countsMap) | broad.pda.seq.segmentation.ContinuousDataAlignmentModel |  |
| filterPaths(Map< Path, double[]> scores, double alpha2) | broad.pda.seq.segmentation.ContinuousDataAlignmentModel | static |
| getAlignmentDataModelStats() | broad.pda.seq.segmentation.ContinuousDataAlignmentModel |  |
| getBasesCovered(Annotation region) | broad.pda.seq.segmentation.ContinuousDataAlignmentModel |  |
| getBasesCovered(Annotation region, int EF) | broad.pda.seq.segmentation.ContinuousDataAlignmentModel |  |
| getChromosomeLength(String chr) | broad.pda.seq.segmentation.ContinuousDataAlignmentModel |  |
| getChromosomeLengths() | broad.pda.seq.segmentation.ContinuousDataAlignmentModel |  |
| getCount(Collection< Annotation > Annotation) | broad.pda.seq.segmentation.ContinuousDataAlignmentModel |  |
| getCounts(Map< String, IntervalTree< Annotation >> geneTree, Collection< String > multimappers) | broad.pda.seq.segmentation.ContinuousDataAlignmentModel |  |
| getCountsPerBp(Annotation align, IntervalTree< Alignment > tree) | broad.pda.seq.segmentation.ContinuousDataAlignmentModel |  |
| getCountsPerBp(Gene gene, IntervalTree< Alignment > tree) | broad.pda.seq.segmentation.ContinuousDataAlignmentModel |  |
| getData() | broad.pda.seq.segmentation.ContinuousDataAlignmentModel |  |
| getDataForAlignment(Annotation align) | broad.pda.seq.segmentation.ContinuousDataAlignmentModel |  |
| getDataForGene(Gene gene) | broad.pda.seq.segmentation.ContinuousDataAlignmentModel |  |
| getDataForGene(Collection< Gene > Annotation) | broad.pda.seq.segmentation.ContinuousDataAlignmentModel |  |
| getGeneCounts(Collection< Gene > genes) | broad.pda.seq.segmentation.ContinuousDataAlignmentModel |  |
| getGeneCountsWithoutDatasetTotals(Collection< Gene > genes) | broad.pda.seq.segmentation.ContinuousDataAlignmentModel |  |
| getGeneExpression(Collection< Gene > genes) | broad.pda.seq.segmentation.ContinuousDataAlignmentModel |  |
| getLambda(String chr) | broad.pda.seq.segmentation.ContinuousDataAlignmentModel |  |
| getNumberMarkers(String chr) | broad.pda.seq.segmentation.ContinuousDataAlignmentModel |  |
| getNumberOfBPs() | broad.pda.seq.segmentation.ContinuousDataAlignmentModel |  |
| getNumberOfReads(String chr) | broad.pda.seq.segmentation.ContinuousDataAlignmentModel |  |
| getPairedData() | broad.pda.seq.segmentation.ContinuousDataAlignmentModel |  |
| getPeak(Gene gene, Gene startCodon, IntervalTree< Alignment > tree) | broad.pda.seq.segmentation.ContinuousDataAlignmentModel |  |
| getPeak(Gene gene, Gene startCodon) | broad.pda.seq.segmentation.ContinuousDataAlignmentModel |  |
| getSum(String chr) | broad.pda.seq.segmentation.ContinuousDataAlignmentModel |  |
| getWidths(String str) | broad.pda.seq.segmentation.ContinuousDataAlignmentModel | static |
| hasDataForChromosome(String chr) | broad.pda.seq.segmentation.ContinuousDataAlignmentModel |  |
| main(String[] args) | broad.pda.seq.segmentation.ContinuousDataAlignmentModel | static |
| parseMaskFiles(File[] files) | broad.pda.seq.segmentation.ContinuousDataAlignmentModel | static |
| permuteRegions(Annotation align) | broad.pda.seq.segmentation.ContinuousDataAlignmentModel |  |
| rawRegions(Annotation align) | broad.pda.seq.segmentation.ContinuousDataAlignmentModel |  |
| resetTreeCache() | broad.pda.seq.segmentation.ContinuousDataAlignmentModel |  |
| scan(int[] windowSizes, double alpha) | broad.pda.seq.segmentation.ContinuousDataAlignmentModel |  |
| scan(int[] windowSizes, double alpha, String chr) | broad.pda.seq.segmentation.ContinuousDataAlignmentModel |  |
| scanGenome(ContinuousDataAlignmentModel data2, int windowSize, String chr, FileWriter writer, boolean filterSignificance) | broad.pda.seq.segmentation.ContinuousDataAlignmentModel |  |
| scanPRate(Gene gene) | broad.pda.seq.segmentation.ContinuousDataAlignmentModel |  |
| scanPRate(Gene gene, IntervalTree< Alignment > tree) | broad.pda.seq.segmentation.ContinuousDataAlignmentModel |  |
| scanPRate(Gene gene, IntervalTree< Alignment > tree, double localLambda) | broad.pda.seq.segmentation.ContinuousDataAlignmentModel |  |
| scanPRate(Gene gene, IntervalTree< Alignment > tree, double localLambda, int numMarkers) | broad.pda.seq.segmentation.ContinuousDataAlignmentModel |  |
| scanRegion(int fixedWidth, LightweightGenomicAnnotation region) | broad.pda.seq.segmentation.ContinuousDataAlignmentModel |  |
| scoreGene(Gene align) | broad.pda.seq.segmentation.ContinuousDataAlignmentModel |  |
| scoreGene(Gene align, double localLambda) | broad.pda.seq.segmentation.ContinuousDataAlignmentModel |  |
| scoreGene(Gene align, IntervalTree< Alignment > tree, double localLambda) | broad.pda.seq.segmentation.ContinuousDataAlignmentModel |  |
| scoreGene(Gene align, IntervalTree< Alignment > tree, double localLambda, int numMarkers) | broad.pda.seq.segmentation.ContinuousDataAlignmentModel |  |
| scoreGene(Gene align, IntervalTree< Alignment > tree) | broad.pda.seq.segmentation.ContinuousDataAlignmentModel |  |
| scoreGenes(Collection< Gene > set, String chrToUse, IntervalTree< Alignment > tree) | broad.pda.seq.segmentation.ContinuousDataAlignmentModel |  |
| scoreGenes(Collection< Gene > genes) | broad.pda.seq.segmentation.ContinuousDataAlignmentModel |  |
| scoreGenes(Collection< Gene > set, String chrToUse) | broad.pda.seq.segmentation.ContinuousDataAlignmentModel |  |
| scoreGenes(Collection< Gene > set, String chrToUse, int size) | broad.pda.seq.segmentation.ContinuousDataAlignmentModel |  |
| scoreGenes(BEDFileParser genes) | broad.pda.seq.segmentation.ContinuousDataAlignmentModel |  |
| scorePaths(Collection< Path > paths, double lambda) | broad.pda.seq.segmentation.ContinuousDataAlignmentModel |  |
| scorePaths(Collection< Path > paths, ChromosomeWithBubblesJGraphT graph, double alpha) | broad.pda.seq.segmentation.ContinuousDataAlignmentModel | static |
| scorePaths(Collection< Path > paths, double lambda, double numMarkers, double numReads, double RPKMContant, Map< Annotation, Double > localRates, double alpha) | broad.pda.seq.segmentation.ContinuousDataAlignmentModel | static |
| scorePathsForOnlyRPKM(Collection< Path > paths) | broad.pda.seq.segmentation.ContinuousDataAlignmentModel |  |
| scoreSegment(Annotation align) | broad.pda.seq.segmentation.ContinuousDataAlignmentModel |  |
| scoreSegments(Collection< Annotation > set) | broad.pda.seq.segmentation.ContinuousDataAlignmentModel |  |
| scoreSegments(Collection< Annotation > set, String chrToUse) | broad.pda.seq.segmentation.ContinuousDataAlignmentModel |  |
| scoreSegments(Collection< Annotation > set, String chrToUse, boolean ignoreAlpha) | broad.pda.seq.segmentation.ContinuousDataAlignmentModel |  |
| setAlpha(double alpha) | broad.pda.seq.segmentation.ContinuousDataAlignmentModel |  |
| setExtensionFactor(int extensionFactor) | broad.pda.seq.segmentation.ContinuousDataAlignmentModel |  |
| setFindMaxContiguous(boolean findMaxContiguous) | broad.pda.seq.segmentation.ContinuousDataAlignmentModel |  |
| setMaskFileData(Map< String, Integer > maskedRegionMap) | broad.pda.seq.segmentation.ContinuousDataAlignmentModel |  |
| setMinContguousSegmentSize(int minSize) | broad.pda.seq.segmentation.ContinuousDataAlignmentModel |  |
| setMinimumMappingQuality(double minMapQual) | broad.pda.seq.segmentation.ContinuousDataAlignmentModel |  |
| setMinNumberOfSplices(int minSpliceSupport) | broad.pda.seq.segmentation.ContinuousDataAlignmentModel |  |
| setTrimEnds(boolean trimEnds) | broad.pda.seq.segmentation.ContinuousDataAlignmentModel |  |
| setTrimQuantile(double quantile) | broad.pda.seq.segmentation.ContinuousDataAlignmentModel |  |
| setUpWeightSplices(boolean setFlag) | broad.pda.seq.segmentation.ContinuousDataAlignmentModel |  |
| trimGeneEnds(Gene gene, double quantile) | broad.pda.seq.segmentation.ContinuousDataAlignmentModel |  |
| trimMaxContiguous(Gene region, double quantile) | broad.pda.seq.segmentation.ContinuousDataAlignmentModel |  |
| writeConnections(String save, ChromosomeWithBubblesJGraphT graph) | broad.pda.seq.segmentation.ContinuousDataAlignmentModel |  |
| writeFullBED(String save, Map< Gene, double[]> map, double alpha) | broad.pda.seq.segmentation.ContinuousDataAlignmentModel | static |
| writeFullBED(String save, Collection< Gene > segments) | broad.pda.seq.segmentation.ContinuousDataAlignmentModel | static |


---

Generated on Fri Aug 15 2014 15:11:04 for ESAT by  

 1.8.7
